# Acute partial sleep restriction does not impact arterial function in young and healthy humans

**DOI:** 10.1113/EP091699

**Published:** 2024-06-20

**Authors:** Joshua M. Cherubini, Jem L. Cheng, Calvin M. Armstrong, Michael J. Kamal, Gianni Parise, Maureen J. MacDonald

**Affiliations:** ^1^ Department of Kinesiology McMaster University Hamilton Ontario Canada

**Keywords:** cardiorespiratory fitness, circadian rhythms, sleep restriction, vascular dynamics

## Abstract

Habitual short sleep durations are associated with several cardiovascular diseases. Experimental research generally supports these findings as metrics of arterial function are impaired after complete deprivation of sleep and after longer periods of partial sleep restriction. The acute influence of a single instance of partial sleep restriction (PSR), however, has not been defined. We evaluated arterial structure and function among 32 university‐aged participants on two occasions: once after normal habitual sleep (NS), and again the morning after an acute partial sleep restriction (PSR) intervention involving only 3 h of sleep for a single night. Endothelial function was measured using ultrasonography at the brachial artery via flow‐mediated dilatation (FMD), and a ramp peak oxygen uptake test was used to evaluate cardiorespiratory fitness. Blood samples were collected from a subset of participants to investigate the influence of circulatory factors on cellular mechanisms implicated in endothelial function. Sleep duration was lower after a night of PSR compared to NS (*P *< 0.001); however, there were no appreciable differences in any haemodynamic outcome between conditions. FMD was not different between NS and PSR (NS: 6.5 ± 2.9%; PSR: 6.3 ± 2.9%; *P* = 0.668), and cardiorespiratory fitness did not moderate the haemodynamic response to PSR (all *P *> 0.05). *Ex vivo* cell culture results aligned with in vivo data, showing that acute PSR does not alter intracellular processes involved in endothelial function. No differences in arterial structure or function were observed between NS and acute PSR in healthy and young participants, and cardiorespiratory fitness does not modulate the arterial response to acute sleep restriction.

## Abstract (highlights)

- **What is the central question of the study?**
  Does a single night of restricted sleep alter indices of arterial structure and function? and if so what is the extent to which cardiorespiratory fitness might modulate the relationship between acute sleep perturbation and vascular function in humans?
- **What is the main finding and its importance?**
  One night of partial sleep restriction does not affect arterial function or structure in young and healthy humans, and arterial function may be sensitive to prolonged, but not necessarily acute, reductions in sleep durations.

## 1 INTRODUCTION

Mendelian randomization studies show causal associations between genetically predicted habitual short sleep durations and several cardiovascular diseases (Ai et al., 2021; Daghlas et al., 2019). These findings accord with results from experimental interventions wherein short sleep durations impair endothelial function in both the microvasculature and macrovasculature (Holmer et al., 2021). That endothelial dysfunction is an antecedent to overt cardiovascular disease and occurs early in the atherogenic process (Celermajer et al., 1992) highlights an involvement of sleep duration as an early, and potentially modifiable, step in the progression of cardiovascular diseases. Experimentally induced and chronic short sleep durations involving either the complete or partial deprivation of sleep are consistently associated with impaired endothelial function, including reductions in brachial artery (BA) flow‐mediated dilatation (FMD). However, the acute impact of partial sleep restriction (PSR) on resting arterial function has not been described. Acute PSR, in contrast to total sleep deprivation or chronic PSR, involves just a single night of restricted sleep and is a common occurrence in young and healthy humans (Lund et al., 2010). The influence of repeated instances of acute PSR, even with adequate rest interspersed may accumulate to accelerate atherogenic adaptations in the human vasculature. Increases in inflammatory signaling (Irwin et al., 2006), decreases in the capacity for inflammatory resolution (Engert et al., 2023), augmented cardiovascular reactivity (Yang et al., 2012), and perturbed cardiac chronotropy (Schlagentweit et al., 2023) may all contribute to atherogenesis during repeated bouts of acute PSR. In addition to perturbing cardiovascular control, acute PSR may furthermore pose a methodologically relevant consideration to the assessment of arterial function as sleep durations are not considered in the recommended guidelines for the assessment of endothelial function (Thijssen et al., 2019). Acute PSR may thus be a particularly ecologically relevant sleep restriction intervention to elucidate insight into incipient atherogenic adaptations that begin with short sleep durations when iterated over time.

Several mechanisms underlying associations between insufficient sleep and arterial dysfunction have been proposed. Accelerated haematopoiesis and ensuing monocytosis and inflammation (Irwin et al., 2016; McAlpine et al., 2022, 2019) may decrease the bioavailability of atheroprotective compounds such as nitric oxide (NO) in the endothelium during maladaptive sleep. Indeed, increases in endothelial inflammation, concomitant with decreases in endothelial antioxidant defense capacity, have been reported in sleep‐deprived women (Shah et al., 2018, 2022). While NO bioavailability in murine models is reduced after rapid eye movement (REM)‐specific sleep deprivation (Jiang et al., 2017), there is a paucity of research examining the impact of sleep restriction on NO bioavailability in humans. Given this existing evidence, it stands to reason that circulating factors may modulate NO bioavailability in the endothelium in concert with sleep restriction. We (Cherubini et al., 2021) and others (Holmer et al., 2021) have further postulated that changes in intracellular circadian dynamics may alter endothelial function during sleep deprivation as a result of the sustained exposure to potential stimuli including light, food and activity during sleep restriction. Several circadian genes influence canonical pathways involved in the maintenance of endothelial function (Shang et al., 2016). Changes in these transcription factors in the vascular endothelium, such as those observed in skeletal muscle after sleep restriction (Cedernaes et al., 2015), may have corresponding consequences on endothelial function. Importantly, impairments in the microvasculature induced during acute total sleep deprivation are reversed with exercise training (Sauvet et al., 2017). This suggests the potential for fitness to moderate associations between sleep and vascular function.

We therefore identified three objectives for this investigation. First, the impact of acute PSR on vascular endothelial function and resting haemodynamics in humans was evaluated. Second, we analysed the potential moderating effects of cardiorespiratory fitness given the associations that exist between improved cardiorespiratory fitness and cardiovascular adaptations (Han et al., 2022). Last, an exploratory *ex vivo* cell culture model was used to investigate a mechanistic basis for circulatory factors to affect intracellular endothelial circadian dynamics during acute PSR.

## 2 METHODS

### 2.1 Ethical approval and sample size determination

Young and healthy participants between the ages of 18 and 35 were recruited for this study. Prospective participants were excluded if they were smokers, had a history of cardiovascular, metabolic or musculoskeletal diseases, reported any diagnosed sleeping disorders during medical screening, or reported consumption of medications that would interact with arterial vasomotion. All participants provided informed consent to the experimental procedures, which were approved by the Hamilton Integrated Research Ethics Board (Project ID 8270). Data collection protocols described herein were conducted in accordance to the guidelines established by the *Declaration of Helsinki*, except for registration in a database.

We calculated, with an acceptable type 1 error rate set to 5% and type 2 error rate set to 20%, that 32 participants were required to reliably detect a 0.9% change (Cohen's *d*_z_ = 0.54) in our primary outcome measure, BA FMD, assuming a common standard deviation of 2.2% between sleep conditions. This reflects a medium‐sized change in endothelial function research (Cherubini & MacDonald, 2021). The sample size was determined using the ‘Superpower’ R package (Lakens & Caldwell, 2021) and based on 10,000 simulations with a correlation coefficient set to *r* = 0.7 to account for the repeated measures in our experimental design. We deemed a 0.9% change in FMD to be the smallest effect of interest given that prior literature has associated a 1% increase in relative FMD with an approximately 13% decrease in risk of cardiovascular events (Inaba et al., 2010). All power analyses were performed using two‐tailed models to reliably detect changes in either the positive or negative direction, since the potential for bidirectional changes in endothelial function has been reported after sleep restriction in humans (Sauvet et al., 2010; Yang et al., 2021).

### 2.2 Experimental protocol

Upon arrival to the laboratory, participants were fitted with single‐lead ECG and rested for 10 min in the supine position. Resting haemodynamic measures including systolic blood pressure, diastolic blood pressure, mean arterial pressure and heart rate were subsequently evaluated using a semi‐automated oscillometric blood pressure machine (Dinamap Carescape V100; GE Healthcare, Mississauga, ON, Canada) measured in triplicate. Participants were instructed to arrive in a fasted state for at least 6 h, while abstaining from caffeine and alcohol for at least 12 h, and from any moderate‐to‐vigorous physical activity for 24 h prior to data collection. Female participants were tested during menses, except for one participant who reported use of an intrauterine device. Sleep duration, measured by hours slept, was quantified using three different methods to obtain a comprehensive view of the participants’ sleep. These methods included the use of a consensus sleep diary (Carney et al., 2012) for a subjective account of sleep duration, a Fitbit accelerometer (Charge HR, 2014; San Francisco, CA, USA), and a mobile audio‐recording application, Sleep Cycle (Sleep Cycle, Gothenburg, Sweden) for objective data acquisition. Participants were asked to complete the consensus sleep diary each morning upon waking, and to wear the Fitbit for the duration of their involvement in the study. Participants were also asked to commence mobile audio‐recording upon sleep initiation and terminate the recording upon waking. The total sleep time provided by the Fitbit and mobile audio‐recording application were interpreted as an estimate of sleep duration.

**FIGURE 1:**
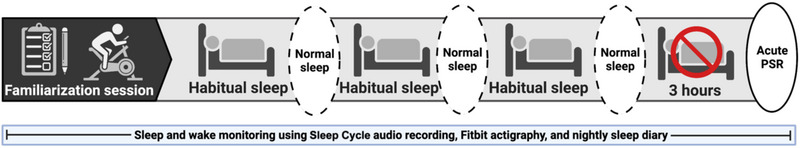
Depiction of the in vivo experimental timeline. Participants arrived at the laboratory for one familiarization visit and two data collection sessions. The first data collection session occurred after one, two, or three nights of habitual sleep. The second data collection session occurred after one night of acute partial sleep restriction where sleep duration was restricted to 3 h. PSR, partial sleep restriction. Figure created using BioRender.com.

The experimental timeline is presented in Figure 1. Participants completed a familiarization visit wherein they were acquainted with experimental procedures and participated in a ${\dot{V}}_{O_{2}}$ peak exercise test. Participants then experienced three nights of normal sleep (NS condition) where they were instructed to continue with their habitual sleeping patterns. Participants attended a vascular testing session in the morning following one of the three nights of normal sleep to minimize any potential order effects. On the fourth night, sleep was partially restricted (PSR condition) to 3 h in the latter half of the night (after 02.00 h). We chose a 3‐h sleep opportunity in the second half of the night to extend data from previous research (Irwin et al., 2006) describing increases in inflammatory signalling after acute PSR in similar experimental interventions. No further sleeping instructions, such as considerations to light exposure, activity or food consumption were given apart from the standardized pre‐visit instructions stated above prior to data collection. Testing sessions for each participant were scheduled at approximately the same time of day to preclude a diurnal influence on vascular function (Jones et al., 2010).

### 2.3 Endothelial function

BA FMD is an indicator of endothelial function and a surrogate for invasive measures of coronary artery endothelial function (Takase et al., 1998). BA FMD was measured at the right brachial artery for all participants while adhering to published guidelines for the standardized assessment of BA FMD (Thijssen et al., 2019). A 12 MHz linear array ultrasound probe was connected to an ultrasound unit (Vivid Q; GE Medical Systems, Horten, Norway) to visualize the brachial artery. The duplex mode was used to obtain concomitant measures of arterial diameter (mm) and mean blood velocity (cm/s). The insonation angle was corrected to 68°. A 30‐s image of the brachial artery was acquired to measure resting artery diameter and blood flow. After the acquisition of the resting images, a pneumatic pressure cuff that had been placed approximately 2–3 cm distal to the medial epicondyle was inflated to suprasystolic pressure equal to 200 mmHg using a rapid inflator (E20 Rapid Cuff Inflator; AG 101 Cuff Inflator Air Source; Hokanson, Bellevue, WA, USA). A 5‐min suprasystolic cuff inflation period was used in accordance with previously published guidelines and standardized assessment of BA FMD (Thijssen et al., 2019). Continuous images of the BA were acquired following cuff deflation for 3 min. All brightness‐mode images were saved as a digital imaging and communications in medicine file, and end‐diastolic frames were extracted and collated for edge‐tracking analysis (Sante DICOM Editor, Version 2.1.20; Santesoft, Athens, Greece). Arterial diameter was measured as the distance (mm) between the near and far arterial wall, including the intima and arterial lumen, using a semi‐automated arterial wall tracking program (Arterial Measurement System Image and Data Analysis; Thomas Gustavsson, Chalmers University of Technology, Sweden). FMD was calculated as: $\%\mathrm{FMD}\;=(\frac{\mathrm{Peak}\;\mathrm{AD}\;-\mathrm{Baseline}\;\mathrm{AD}}{\mathrm{Baseline}\;\mathrm{AD}})\;\times \;100$.

Allometric scaling of the brachial artery FMD response was calculated using the ‘Rtery’ package. The diameter change was fit to a linear mixed effects model that measured log‐peak diameter as a function of log‐baseline diameter, with participants coded as a random factor to account for the correlated measures within each participant. Estimated marginal means and measures of variance were back‐transformed to obtain estimates of arterial diameter changes scaled to baseline arterial diameter.

Shear rate (s^−1^) was calculated as a surrogate for shear stress, given the absence of blood viscosity data to inform shear stress. We used the formula $\mathrm{SR}\;=\frac{8\;\times \;\mathrm{MBV}\;}{\mathrm{AD}}\;$ to calculate shear rate (SR), where MBV represents the mean blood velocity (cm/s) and AD represents arterial diameter. The shear rate area under the curve until peak dilatation was interpreted as the relevant dilatory stimulus (Pyke & Tschakovsky, 2007).

### 2.4 Central pulse wave velocity

Central pulse wave velocity between the carotid and femoral arteries (cfPWV) was assessed using applanation tonometry. cfPWV was measured in accordance with the evidence‐based guidelines for the acquisition of central arterial stiffness (van Bortel et al., 2012) to evaluate changes in arterial stiffness after acute PSR. Pressure from one hand‐held tonometer was applied distal to the carotid sinus and a second tonometer was simultaneously applied to the common femoral artery in the inguinal region. Pressure waveforms derived from micromanometer‐tipped tonometers (model SPT‐301; Millar Instruments Inc., Houston, TX, USA) were gated to ECG tracings and passed through an analog‐to‐visual acquisition and converting unit (PowerLab 16/30 displayed on LabChart8 Pro; ADInstruments, Inc., Colorado Springs, CO, USA). Signals were band‐pass filtered with a high cut‐off frequency set to 30 Hz and a low cut‐off frequency set to 5 Hz. The straight distance between applanation sites over the surface of the body was calculated as the average of two consecutive measures using a standard anthropometric tape measure, and 80% of this distance was calculated and used to determine cfPWV.

### 2.5 Heart rate variability

Resting heart rate variability (HRV) provides an indirect measure of cardiovagal input and parasympathetic nervous system influence (Camm et al., 1996). Participants were fitted with single‐lead electrocardiography to collect continuous cardiac signals. The cardiac ECG data were recorded at a sampling frequency of 1000 Hz for 5 min at the beginning of each data collection session while participants were in the supine position. Only high‐frequency activity could be estimated from this recording duration. HRV was therefore represented in the time‐domain as the root‐mean square of successive RR intervals (RMSSD; m/s) using algorithms provided by LabChart8 Pro (ADInstruments Inc.) analysis software.

### 2.6 ${\dot{V}}_{O_{2}\mathrm{peak}}$ testing

Cardiorespiratory fitness was estimated using an incremental exercise test to exhaustion. Participants were fitted with a heart rate (HR) monitor (Model A300, Polar H9 heart rate sensor; Polar Electro Oy, Kempele, Finland) and face mask for a ramp exercise protocol on a stationary cycle ergometer. The test started with a 3‐min warm‐up at 50 W. Due to a technical malfunction of the cycle ergometer during the experiment, we used two ramp protocols after warm‐up including (i) increases in work at a rate of 1 W every 2 s thereafter, or (ii) at a rate of 5 W every 10 s after the warm‐up. Participants pedalled until their cadence dropped below 60 rpm or until volitional exhaustion. Further criteria for obtaining a ${\dot{V}}_{O_{2}\mathrm{peak}}$ included a plateau in O_2_ consumption despite increases in work, reaching age‐predicted HR max, and demonstrating a respiratory exchange ratio ≥1.1 (Howley et al., 1995). The average of the largest three consecutive relative ${\dot{V}}_{O_{2}}$ values recorded in 10 s bins was interpreted as ${\dot{V}}_{O_{2}\mathrm{peak}}$ and was reported relative to body mass (mL/kg/min).

### 2.7 Venous blood sampling extraction and processing

Venous blood samples were taken from the antecubital vein of a subset of nine participants from whom blood samples were available. Samples were collected immediately following blood pressure measurements after the supine resting period, and prior to assessments of arterial structure and function. Whole blood was collected into 4.0 mL BD vacutainer tubes for serum (BD Vacutainer; BD Biosciences, San Jose, CA, USA), inverted several times immediately after extraction, and then allowed to rest at room temperature for 45 min before centrifugation for 10 min at 4°C at 4000 r.p.m. The serum supernatant was collected, and all samples were stored at −80°C until use. We deemed nine participants an appropriate sample size for these exploratory analyses given that previous research with a similar or fewer number of samples captured modest effects of serum exposure (Brunt et al. 2019) or circadian dynamics (Mastrullo et al., 2022) in human umbilical vein endothelial cells (HUVEC).

### 2.8 Exploratory analyses: *ex vivo* HUVEC culture preparations

We performed additional experiments to explore the effects of the circulatory milieu following sleep restriction on intracellular mechanisms involved in endothelial function. All cell culture experiments were performed using HUVECs (PCS‐100‐013; ATCC, Manassas, VA, USA) between passage 5 and 7. HUVECs were cultured in complete endothelial cell growth medium (ECGM; PromoCell, Heidelberg, Germany) supplemented with 2% fetal bovine serum (FBS; Thermo Fisher Scientific, Waltham, MA, USA), 1% penicillin streptomycin (Thermo Fisher Scientific), and 2.52% supplement mix (PromoCell). Some research (Takeda et al., 2007), but not others (Mastrullo et al., 2022), has shown rhythmicity of circadian genes in cultured HUVECs. Therefore, prior to all experiments, HUVECs were cultured in serum‐free ECGM supplemented with 50% FBS (Thermo Fisher Scientific) for 2 h at 5% CO_2_ and 37°C, per established FBS‐shock protocol (Balsalobre et al., 1998). This was done to synchronize the circadian phase between cells and minimize the heterogeneity of intercellular differences in circadian time. Experiments were performed when cells reached approximately 100% confluence for several reasons. First, we wanted to recapitulate the in vivo environment where endothelial cells form a confluent monolayer along the vascular lumen. Second, endothelial cells engage in contact‐dependent cell cycle exit into the quiescent G_0_ phase (Herbert et al., 2009). This is advantageous because it allowed us to ensure all intracellular circadian components could be synchronized during the FBS shock, and preclude the potential for the dampening of amplitude or phase that might occur during mitosis in the absence of synchronizing stimuli (Mastrullo et al., 2022). Two hours after FBS shock, cells were washed with sterile phosphate‐buffered saline (PBS; Thermo Fisher Scientific) before being cultured with 10% human serum collected after both NS and acute PSR, prepared in ECGM.

### 2.9 Nitric oxide and bulk reactive oxygen species imaging

We sought to examine differences in NO bioavailability, NO expression during acetylcholine (ACh) provision and reactive oxygen species (ROS) among cells cultured in serum collected following the sleep restriction condition. For all imaging experiments, HUVECs were plated in 96‐well plates and allowed to adhere for at least 48 h with routine medium changes prior to FBS shock. Cells were then cultured with ECGM supplemented with 10% human serum from participants after NS or acute PSR. At the indicated times post‐FBS shock, the spent medium was aspirated and the cells were washed twice with sterile Hank's balanced salt solution (HBSS). To fluorescently label NO, 10 μM of 4‐amino‐5‐methylamino‐2′,7′‐difluorofluorescein diacetate (DAF‐FM; Cayman Chemical Company, Ann Arbor, MI, USA) dissolved in basal ECGM was added to each well. Cells were incubated with DAF‐FM and ECGM in a 37°C, 5% CO_2_ incubator for 30 min. The cells were then washed twice with HBSS and immediately imaged at ×20 magnification with an Eclipse Ti fluorescence microscope (Nikon Instruments, Mississauga, ON, Canada). To examine the HUVEC capacity to produce NO after being cultured with NS or PSR serum, ACh (200 μM) was added to each well and images were captured at 5 and 20 min after ACh provision as previously described (Craighead et al., 2021; LaRocca et al., 2012). The HUVEC response to ACh was calculated as the fold change from basal condition. To examine bulk ROS, 5 μM of a fluorescent probe, CellROX (Thermo Fisher Scientific), was dissolved in ECGM and added to the HUVECs. Cells were again incubated with CellROX and ECGM in a 37°C, 5% CO_2_ incubator for 30 min. The cells were washed twice using HBSS and then immediately imaged at ×20 magnification with an Eclipse Ti2 fluorescence microscope (Nikon Instruments). For both DAF‐FM and CellROX staining, HUVEC nuclei were labelled with Hoechst 33342 solution.

### 2.10 RNA isolation, cDNA synthesis and quantitative PCR

Serum‐exposed HUVECs were collected at 6, 12, 18 and 24 h post‐FBS shock for quantitative PCR (qPCR) experimentation. RNA was isolated using the ENZA Total RNA Isolation Kit (Omega Biotek, Norcross, GA, USA) as per the manufacturer's instructions, and total RNA concentration was measured with a Nanodrop 1000 spectrophotometer (Thermo Fisher Scientific). cDNA was synthesized using the high‐capacity cDNA synthesis Kit (Thermo Fisher Scientific). Amplification of our genes of interest involved in circadian regulation (*CLOCK*, *BMAL1*, *PER2*, *CRY2*) and endothelial function (*NOS3* and *AKT1*) was performed with TaqMan assays, and mRNA expression was quantified using the comparative $2^{-\Delta \Delta C_{t}}$ method and normalized to the *GAPDH* reference gene (Livak & Schmittgen, 2001). *C*_t_ values were obtained in triplicate and a no‐template control was utilized to ensure the absence of non‐specific amplification. TaqMan assays were purchased from Thermo Fisher Scientific and the assay details are included in Table 1.

**TABLE 1:** TaqMan assays used for qPCR.

Gene name	Gene	TaqMan assay ID
Nitric oxide synthase 3	*NOS3*	Hs01574659_m1
AKT serine/threonine kinase 1	*AKT1*	Hs00178289_m1
Circadian locomotor output cycles protein kaput	*CLOCK*	Hs00231857_m1
Basic helix–loop–helix ARNT like 1	*BMAL1*	Hs00154147_m1
Period circadian regulator 2	*PER2*	Hs00256143_m1
Cryptochrome circadian regulator 2	*CRY2*	Hs00323654_m1

### 2.11 Statistical methods

All statistical treatments were performed in the R environment (Version 4.1.0; R Core Team, 2013). The materials required to support our analyses are publicly available and deposited in the Borealis open‐access repository.

The influence of sleep restriction on in vivo indices of haemodynamics, endothelial function, arterial stiffness and HRV was assessed using a paired Welch's *t*‐test. To evaluate the influence of cardiorespiratory fitness on the aforementioned relationships, we added ${\dot{V}}_{O_{2}\mathrm{peak}}$ as a moderating variable into a linear mixed effects model that allowed intercepts to vary by participant, read FMD ∼ Condition × ${\dot{V}}_{O_{2}\mathrm{peak}}$ + (1 | Participant ID), using formulae defined by Montoya (2019). This syntax facilitated the incorporation of ${\dot{V}}_{O_{2}\mathrm{peak}}$ as an interaction variable to measure the extent to which changes in endothelial function were moderated by cardiorespiratory fitness. An absence of evidence for an effect of acute PSR on FMD should not be interpreted as evidence supporting no true effect (Lakens, 2017). Therefore, a two one‐sided test for equivalence (‘TOSTER’ package; Caldwell, 2022; Mirabella et al., 2017) was used when we deemed it theoretically important to substantiate the absence of an effect for non‐significant results (*P* ≥ 0.05). We set equivalence bounds according to the small effect that our a priori power calculation had 80% power to detect in either direction: −0.9 ≤ ΔFMD(%) ≤ 0.9.

The package ‘Circacompare’ was used to detect rhythmicity and statistically compare groups of rhythmic data (Parsons et al., 2020). For rhythmic components (*y*), a non‐linear mixed‐effects regression model of the form y=k+α×cos(t−φ) was used to statistically compare rhythmic parameters including amplitude (α), acrophase (φ), and the rhythm‐adjusted mean (*k*) of each curve across time (*t*), as per Parsons et al. (2020). This analysis generates an estimate for the aforementioned rhythmic parameters of interest and an estimate for the difference in a rhythmic parameter between two groups of rhythmic data. If data from either NS or PSR condition revealed an arrhythmic pattern, a repeated measure two‐way ANOVA (condition [2 levels: NS and PSR] × time [4 levels: 6, 12, 18, and 24 h]) was used to detect differences between conditions. All tests were two‐tailed with the rate of type 1 errors set at α = 0.05. Data is shown as means ± standard deviation (SD), and means ± standard error (SE) with 95% confidence intervals for rhythmic parameters unless stated otherwise.

## 3 RESULTS

Data from a total of 32 participants (16 M; 16F) are included in this study. An anthropometric description of the participant sample is provided in Table 2. Data from female participants were collected during the early follicular phase (days 1–7 of the menstrual cycle) as determined by self‐report (naturally cycling: *n* = 11; oral contraceptive pill: *n* = 4), except for one participant who reported use of an intrauterine device. Data after NS were collected at approximately 10:35 ± 02:06 (hh:mm) and data after acute PSR were collected at approximately 10:34 ± 02:03 hh:mm. Importantly, the assessment time of day was kept approximately the same within participants (time difference between visits: 00:19 ± 00:22 hh:mm). Sleep duration, whether estimated using the Fitbit device, Sleep Cycle audio recording application, or self‐report diary, was significantly reduced between normal sleep and acute partial sleep restriction across all modalities (all *P* < 0.001) as per our intended experimental intervention (Table 3).

**TABLE 2:** Demographic description of the research participants.

Variable	Value (*n* = 32)
Sex	16 M/16F
Age (years)	21 ± 3
Habitual self‐reported sleep duration (h)	7.4 ± 0.8
Habitual self‐reported bedtime (hh:mm)	00:06 ± 01:10
Height (m)	1.71 ± 0.1
Weight (kg)	69.7 ± 10.4
BMI (kg/m^2^)	23.9 ± 3.0
${\dot{V}}_{O_{2}\mathrm{peak}}$ (mL/kg/min)	41.7 ± 8.5

*Note*: Data are shown as means ± SD. BMI, body mass index.

**TABLE 3:** Sleep parameters and resting haemodynamics measured after normal sleep and after acute partial sleep restriction.

Variable	Normal sleep	Partial sleep restriction	*P*	Interaction model estimate β ± SE	Interaction model *P*
Fitbit sleep duration (h)	6.4 ± 2.1	2.7 ± 0.7	**<0.001**	0.01 ± 0.05	0.822
Sleep Cycle sleep duration (h)	6.4 ± 1.1	2.4 ± 0.7	**<0.001**	0.01 ± 0.02	0.585
Self‐report sleep duration (h)	7.2 ± 0.6	2.9 ± 0.4	**<0.001**	0.03 ± 0.01	**0.029**
SBP (mmHg)	114 ± 11	114 ± 11	0.928	0.17 ± 0.09	0.064
DBP (mmHg)	63 ± 6	62 ± 5	**0.048**	0.04 ± 0.07	0.566
MAP (mmHg)	82 ± 7	81 ± 6	0.122	0.09 ± 0.06	0.147
PP (mmHg)	50 ± 10	52 ± 11	0.142	0.13 ± 0.10	0.215
HR (bpm)	63 ± 10	63 ± 12	0.983	−0.26 ± 0.13	0.054
RMSSD (ms)	61.3 ± 39.4	64.0 ± 47.1	0.620	0.31 ± 0.66	0.645
Resting AD (mm)	3.56 ± 0.49	3.55 ± 0.54	0.529	<0.01 ± < 0.01	0.660
Peak AD (mm)	3.78 ± 0.48	3.76 ± 0.51	0.293	<0.01 ± < 0.01	0.912
Abs FMD (mm)	0.22 ± 0.09	0.22 ± 0.09	0.503	<0.01 ± < 0.01	0.610
SR_AUC_ (10^4^) to peak diameter	3.68 ± 1.89	3.39 ± 1.74	0.210	22.64 ± 29.98	0.456

*Note*: Table description: Data are expressed as mean ± SD. Sample *n* = 32 for all measures except Fitbit *n* = 28 and SleepCycle *n* = 26 due to technical difficulty or participant compliance with sleep modalities. Bold font indicates statistical significance (*p* < 0.05). Abbreviations: AD, arterial diameter; Abs FMD, absolute flow‐mediated dilatation; DBP, diastolic blood pressure; FMD, flow‐mediated dilatation; HR, heart rate; MAP, mean arterial pressure; PP, pulse pressure; RMSSD, root‐mean square of successive RR intervals; SBP, systolic blood pressure; SR, shear rate.

### 3.1 Resting haemodynamics were unaffected by acute partial sleep restriction

We wanted to investigate the effects of acute PSR on central haemodynamics including blood pressure and heart rate. We found no appreciable effects of sleep restriction on any haemodynamic variable. Resting heart rate was unchanged between conditions (NS: 63 ± 10, PSR: 63 ± 12 bpm; *P* = 0.983), as was systolic blood pressure (NS: 114 ± 11, PSR: 114 ± 11 mmHg; *P* = 0.928) and mean arterial pressure (NS: 82 ± 7, PSR: 81 ± 6 mmHg; *P* = 0.122). However, diastolic blood pressure was reduced by approximately 1 mmHg after sleep restriction compared to normal sleep (NS: 63 ± 6, PSR: 62 ± 5 mmHg, *P* = 0.048). Because we saw no change in systolic blood pressure (SBP), and a decrease in diastolic blood pressure (DBP), we thought that pulse pressure may increase and reflect an increased demand on the capacity for the microvasculature to buttress the transmural force of arterial blood propagation. However, pulse pressure also remained unchanged (NS: 50 ± 10, PSR: 52 ± 11 mmHg; *P* = 0.142) between the sleep conditions (Table 3). Sex differences have been identified in haemodynamic responses to prolonged sleep restriction in humans (Covassin et al., 2021). We found, however, no sex differences in any of the aforementioned haemodynamics (all *P* > 0.05) measured after NS and acute PSR in our sample of young and healthy humans.

### 3.2 One night of sleep restriction did not impact indices of arterial structure or function

**FIGURE 2:**
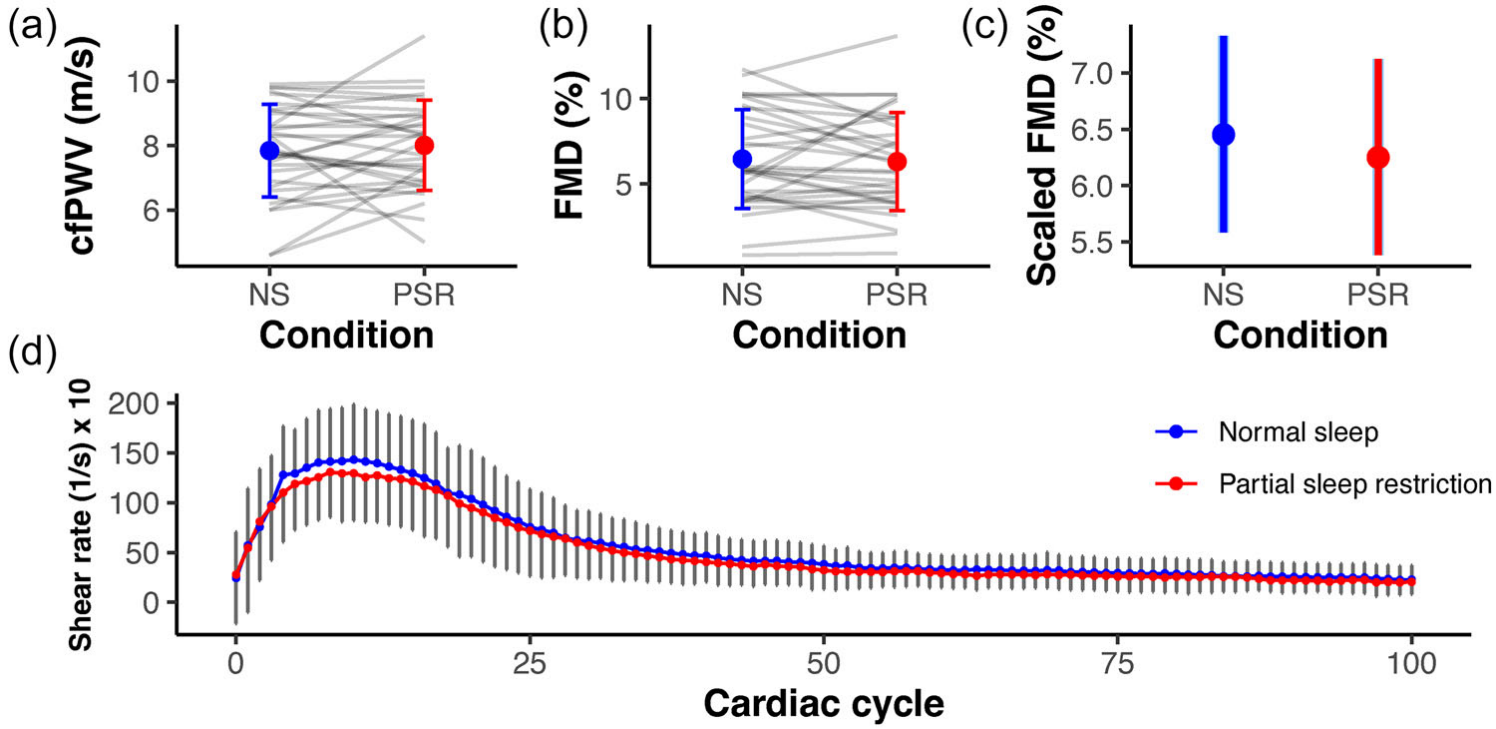
Acute partial sleep restriction did not affect carotid‐femoral pulse wave velocity (cfPWV; a) or arterial function (b, c). Importantly, there was no difference in the shear rate vasodilatory stimulus between the sleep conditions either (d). Graphs show means ± SD with the exception of (c) which shows mean ± 95% CI. NS, normal habitual sleep; PSR, partial sleep restriction.

Acute PSR did not affect measures of arterial structure or function (Figure 2) including brachial artery diameter (NS: 3.56 ± 0.49, PSR: 3.55 ± 0.53 mm, *P* = 0.529), cfPWV (NS: 7.8 ± 1.4, PSR: 8.0 ± 1.4 m/s, *P* = 0.449), or %FMD (NS: 6.5 ± 2.9%, PSR: 6.3 ± 2.9%; *P* = 0.668). The %FMD statistic was allometrically scaled given the propensity for %FMD to be influenced by baseline arterial diameter, and still no significant changes were observed between conditions (back‐transformed mean ± SE; NS: 6.5 ± 0.41%, PSR: 6.2 ± 0.41%, *P* = 0.544). This statistically insignificant relationship also persisted after adjusting for the shear rate area under the curve (AUC) stimulus until peak dilatation (β = −0.01; SE = 0.34; *P* = 0.966). Equivalence testing was performed on %FMD to substantiate the absence of an effect of acute PSR on endothelial function. The null hypothesis of the equivalence test, which tested that the true difference in %FMD was greater than 0.9 or less than −0.9, was rejected (*P* = 0.021). Therefore, the difference in %FMD in the current study may be considered equivalent within our equivalence bounds and provides further evidence that supports a null effect of acute PSR on endothelial function. We again considered sex differences in arterial structure and function given the reported sex‐specific discrepancies in microvascular function after repeated instances of partial sleep restriction in humans (Yang et al., 2021). However, no evidence of sex differences was found with respect to %FMD or cfPWV (all *P* > 0.05) in our sample.

### 3.3 Cardiorespiratory fitness did not moderate the haemodynamic or endothelial function responses to acute partial sleep restriction

**FIGURE 3:**
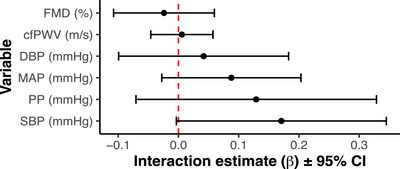
Interaction estimates for the change in the indicated haemodynamic variable before and after sleep restriction and cardiorespiratory fitness estimated by ${\dot{V}}_{O_{2}\mathrm{peak}}$ (mL/kg/min). The point estimate of the interaction (β) is shown along with the 95% confidence interval. None of the representative haemodynamic variables were moderated by ${\dot{V}}_{O_{2}\mathrm{peak}}$, indicated by the 95% CI overlap with the dashed line at 0.0 on the *x*‐axis. PP, pulse pressure.

Participants recorded a mean ${\dot{V}}_{O_{2}\mathrm{peak}}$ of 41.7 ± 8.5 mL/kg/min. No association between any haemodynamic variable and ${\dot{V}}_{O_{2}\mathrm{peak}}$ was found (all *P* > 0.05; Figure 3). Representative relationships are presented in Figure 3 and the β ± standard error of the moderated regression estimates for all variables is provided in Table 4.

**TABLE 4:** mRNA abundance in HUVECs cultured in serum extracted from participants before and after a sleep restriction intervention at 6, 12, 18 and 24 h time points.

						ANOVA *P*		
Gene	Condition	6 h	12 h	18 h	24 h	Interaction	Time	Condition
Nitric oxide provision and endothelial function mRNA abundance								
* NOS3*	NS	1.00 ± 0.34	1.09 ± 0.49	0.72 ± 0.30	0.78 ± 0.25	0.523	0.063	0.079
	PSR	1.03 ± 0.75	1.61 ± 1.37	1.11 ± 0.72	0.73 ± 0.27			
* AKT1*	NS	1.00 ± 1.05	0.56 ± 0.28	0.59 ± 0.55	0.56 ± 0.30	0.647	0.364	0.573
	PSR	0.93 ± 1.00	1.07 ± 1.74	0.62 ± 0.46	0.48 ± 0.19			
Circadian‐related mRNA abundance								
* BMAL1*	NS	1.00 ± 1.25	0.26 ± 0.20	0.32 ± 0.36	0.35 ± 0.25	0.413	0.081	0.520
	PSR	0.86 ± 0.99	0.95 ± 1.72	0.35 ± 0.34	0.24 ± 0.10			
* CLOCK*	NS	1.00 ± 0.92	0.51 ± 0.41	0.39 ± 0.31	0.45 ± 0.33	0.572	**0.028**	0.350
	PSR	1.05 ± 1.24	0.93 ± 1.05	0.54 ± 0.44	0.40 ± 0.25			
* PER2*	NS	1.00 ± 1.58	0.68 ± 0.84	0.40 ± 0.52	0.33 ± 0.28	0.450	0.208	0.530
	PSR	0.71 ± 0.91	1.76 ± 3.32	0.54 ± 0.85	0.18 ± 0.12			
* CRY2*	NS	1.00 ± 1.47	0.53 ± 0.34	0.38 ± 0.21	0.44 ± 0.27	0.632	0.136	0.251
	PSR	1.13 ± 1.62	1.33 ± 1.89	0.63 ± 0.63	0.29 ± 0.08			

*Note*: mRNA data are represented as means ± SD of the fold change normalized to the *GAPDH* reference gene. *n* = 9 per time point. No significant interaction effects were found for any genes of interest involved in either endothelial function or circadian rhythms. Bold indicates statistical significance (*P* < 0.05). NS, normal sleep, PSR, partial sleep restriction.

### 3.4 Abundance of neither nitric oxide nor reactive oxygen species changed in response to serum exposure from normal sleep or acute partial sleep restriction

**FIGURE 4:**
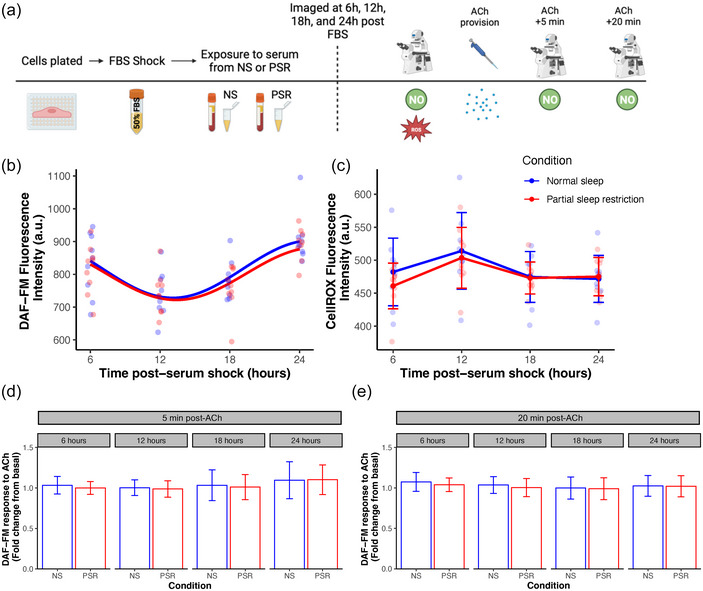
(a) HUVECs were grown in endothelial growth medium, exposed to a serum‐shock (50% FBS) for 2 h, and then cultured in human serum extracted from participants (*n* = 9) after either normal sleep or partial sleep restriction. (b, c) Rhythmic parameters of NO abundance were not affected by serum factors between NS and PSR (b), and ROS abundance also did not change between conditions at any time point (c). (d, e) The fold change in DAF‐FM fluorescence from basal conditions did not change in HUVECs after exposure to the normal sleep or sleep‐restricted circulatory milieu 5 min after ACh provision (d), or 20 min after ACh provision (e). Data show means ± SD.

NO and ROS florescence in HUVEC cultured in serum from a subset (*n* = 9; 5 M/4 F) of participants after normal sleep or PSR was examined at baseline and after ACh provision as a measure of NO production. A weak rhythmic pattern of abundance was detected for DAF‐FM (NS: *P*_rhythm_ < 0.001; PSR: *P*_rhythm_ < 0.001) but not CellROX Deep Red (NS: *P*_rhythm_ = 0.059, PSR: *P*_rhythm_ = 0.076) across a 24‐h period of serum exposure. Rhythmic parameters were statistically compared between the two groups for DAF‐FM fluorescence and no significant differences were observed in the mesor (NS: 815.9 ± 11.9, Δmesor = −14.1 ± 16.8 A.U.; 95% CI: −47.0, 18.9; *P*_k_ = 0.405), amplitude (NS: 87.8 ± 16.8, Δamplitude = −7.8 ± 23.8 A.U., 95% CI: −54.4, 38.8; *P*_α_ = 0.743), or phase (NS: 1.1 ± 0.2, Δphase = 0.1 ± 0.3 rad; 95% CI: −0.5, 0.6; *P*_φ_ = 0.787) between NS and PSR. CellROX fluorescence intensity was highest at 12 h post‐FBS shock (*P*_time_ < 0.001), but no difference in ROS was found between NS and acute PSR at any time point post‐FBS shock (*P*_condition_ = 0.312). ACh induces vascular relaxation via endothelial production of NO (Furchgott & Zawadzki, 1980; Ignarro et al., 1987). Therefore, ACh was added to the HUVEC medium after the acquisition of baseline images, and the DAF‐FM response to ACh was captured after 5 min and again after 20 min of ACh provision. The fold change in DAF‐FM fluorescence relative to baseline at each time point following 5 or 20 min of ACh provision did not change between NS and acute PSR at any time point post‐FBS shock (*P* > 0.05 for all comparisons; Figure 4d).

### 3.5 The expression of canonical genes involved in endothelial function and circadian dynamics remain unchanged after culture with human serum

*NOS3* mRNA expression was found to be weakly rhythmic with a low amplitude for both normal sleep and acute PSR conditions (NS *P*_rhythm_ = 0.017; PSR *P*_rhyhtm_ = 0.035). The rhythmic parameters in *NOS3* abundance did not differ across a circadian period between normal sleep and acute PSR, including no differences in the mesor (NS: 0.9 ± 0.1, Δmesor = 0.2 ± 0.2 A.U.; 95% CI: −0.1, 0.5; *P*_k_ = 0.157), amplitude (NS: 0.2 ± 0.2, Δamplitude = 0.2 ± 0.2 A.U.; 95% CI: −0.2, 0.6; *P*_α_ = 0.286), or phase (NS: 2.4 ± 0.7, Δphase = 0.8 ± 0.8 rad; 95% CI: −0.7, 2.4; *P*_φ_ = 0.309). Furthermore, a similarly weak and low amplitude rhythmic pattern of abundance was found for *BMAL1* (*P*_rhyhtm_ = 0.043; α = 0.3 ± 0.2; 95% CI: 0.02, 0.7) and *CLOCK* (*P*_rhyhtm_ = 0.025; α = 0.3 ± 0.1; 95% CI: 0.1, 0.6) mRNA in HUVECs cultured with serum from the normal sleep condition only. Comparisons in gene expression between NS and PSR remained biologically relevant despite the presence of arrhythmicity in the SD condition. Therefore, data were also compared using a two‐way (2 levels condition × 4 levels time) repeated measures ANOVA to test for differences between group and time. There was no interaction effect for any mRNA involved in endothelial function or any circadian‐related mRNA (all *P* > 0.05; Table 4), and only a main effect of time (*P* = 0.028) for *CLOCK* mRNA. The null in vivo findings were supported at the cellular level as there were no changes in the relative mRNA levels of genes involved in circadian rhythms or endothelial function after serum exposure.

## 4 DISCUSSION

The broad objectives of this study were to characterize the arterial response to acute partial sleep restriction. Acute PSR did not significantly affect macrovascular endothelial function or arterial structure. This finding is conceptually consistent with previous research that also found no significant changes in BA FMD after several repeated bouts of partial sleep restriction lasting three nights at a time (Yang et al., 2021), or in response to high intensity exercise performed after acute PSR (Papadakis et al., 2020). Taken together, we contend that there is no appreciable impact of acute PSR on endothelial function. It is also unlikely that microvascular function (a variable that was not measured herein) changes as it did in Yang et al. (2021) in response to ACh perfusion after sleep restriction, given the lack of changes that were found among any other haemodynamic variable in the current study.

Previous research provides mechanistic support for the notion that acute sleep loss does not affect arterial function, despite our initial supposition that acute PSR would beget atherogenic adaptations. While acute PSR increases tumour necrosis factor and interleukin 6 inflammatory signalling in humans (Irwin et al., 2006), fewer studies report the translation of genetic signalling into circulating peptides during acute instances of maladaptive sleep (Irwin, 2019). Moreover, nuclear factor κB was increased in endothelial cells extracted from participants after 6 weeks of chronic sleep restriction (Shah et al., 2022). The acute nature of our sleep restriction protocol was likely insufficient to result in an atherogenic circulatory milieu and ensuing endothelial dysfunction. Chronic sleep restriction also increases levels of circulating classical monocytes in the evening, but not morning, in humans (McAlpine et al., 2022). Thus, any potential effects of inflammatory monocytes and atherogenic markers on the endothelium may be more pronounced in the evening, instead of in the morning, during which time our data were collected. The endothelium therefore seems to tolerate acute instances of partial sleep restriction, and inflammatory and circadian factors interact to minimize the morning influence of acute maladaptive sleep.

In this study, indices of resting arterial structure and central haemodynamics did not change after acute PSR. The lack of change in BA diameter or cfPWV between conditions supports a null effect of acute instances of PSR on arterial structure. However, indicators of atherosclerosis including ankle‐brachial index and coronary calcium burden are significantly and adversely affected by sleep perturbations to sleep regularity (Full et al., 2023). A role for the irregularity of sleep behaviour, and not necessarily duration (particularly in acute contexts), may be a relevant stimulus for future research to consider in relation to arterial structure and haemodynamics.

There was no moderating effect of cardiorespiratory fitness estimated by relative ${\dot{V}}_{O_{2}\mathrm{peak}}$ (mL/kg/min) on the change in any haemodynamic or vascular function parameter measured after acute PSR. This finding is likely attributable to acute PSR as an insufficient stimulus to cause changes in resting arterial function. Our findings should not preclude the important influence that cardiorespiratory fitness may play in the context of more insulting stimuli, such as in sleep restriction interventions associated with endothelial dysfunction, including total sleep deprivation (Sauvet et al., 2010) and chronic instances of PSR (Covassin et al., 2021; Dettoni et al., 2012; Sauvet et al., 2015). Changes in arterial function during physiological insult have been explained by cardiorespiratory fitness elsewhere (Morishima et al., 2020), and exercise‐trained individuals show attenuated arterial dysfunction during habitual shorter sleep durations (Stockelman et al., 2021). The trivial effects of acute PSR on cardiovascular function described herein may not necessitate the adaptive responses accrued through exercise training. Based on our work and the work of others, we posit that the importance of increased cardiorespiratory fitness on mitigating arterial dysfunction during sleep loss increases as the magnitude of sleep restriction becomes more intense.

The lack of significant changes in macrovascular endothelial function are further supported by our exploratory *ex vivo* cell culture experiments. The exploratory analysis indicated no effect of acute PSR on cellular dynamics involved in endothelial function or circadian rhythms compared to NS. The mostly arrhythmic nature of circadian gene abundance in this study accords with recent work (Mastrullo et al., 2022), and supports the notion that an endogenous circadian system may not be rhythmic in endothelial cells cultured alone or with human serum. In humans, NO‐mediated vasodilatation appears to follow a diurnal pattern (Jones et al., 2010; Otto et al., 2004; Thosar et al., 2019). We found that NO abundance and *NOS3* mRNA were only weakly rhythmic in both normal sleep and sleep restriction conditions, and that the rhythmicity in other mRNA involved in circadian dynamics was variable. These results suggest the influence of other factors, including ROS, circulating factors such as leptin (Mastronardi et al., 2002), or phosphorylated eNOS protein content, to play a more important role in NO bioavailability across a circadian period. Indeed, Jones et al. (2012) indicate that the diurnal variation in brachial FMD is more likely regulated by stochastic factors such as exercise and activity rather than intrinsic endogenous circadian timekeepers. Importantly, however, our exploratory analyses did not suggest differences in any rhythmic or arrhythmic parameter between NS and acute PSR. These exploratory results suggest that, unlike in murine models experiencing paradoxical sleep restriction (Jiang et al., 2017), acute PSR may not result in molecular changes in the endothelium that then require compensation at a physiological systems level. Our results contrast to those observed after chronic sleep restriction in humans (Shah et al., 2022) and indicate that the endothelium is vulnerable to the atherogenic effects of prolonged, but not acute, discontinuous sleep.

### 4.1 Experimental considerations and future directions

Several experimental considerations are important to mention. The exploratory and hypothesis‐generating nature of our *ex vivo* assays renders the results vulnerable to type 2 errors that may lead to a false acceptance of the null hypothesis. Future work may expand upon our results with larger samples. Further, and from a methodological perspective, measures of arterial structure and function after NS always preceded those measures obtained after acute PSR, and investigators were therefore not blinded to the characteristics of the participants’ sleep during data collection. The potential influence of order effects and investigator bias during data acquisition should thus be acknowledged as a relevant experimental consideration. We lacked access to gold‐standard devices to accurately measure sleep, and thus our capacity to accurately quantify sleep duration, and other relevant stimuli associated with sleep such as luminescence, was accordingly limited. Nevertheless, the three devices by which sleep duration was quantified—using accelerometry, mobile audio recording and subjective accounts—provided a combined representation of sleep duration, and all reported a reduction in duration as intended by our intervention. Furthermore, the absence of a sleep laboratory presented the opportunity to simulate the effects of short sleep duration in a relevant setting. Participants could engage in their normal sleeping habits and routines, and thus avoid the influence of stress intrinsic to overnight sleep assessments in an unfamiliar laboratory environment. Moreover, the results of this research are generalizable only to the young and healthy cohort from which data were garnered. These findings cannot be extrapolated to a broader population that may be ageing or living with additional atherosclerotic risk factors. Indeed, the accumulation of atherosclerotic risk factors may magnify any effects of the PSR intervention in such a manner that is consistent with the notion of PSR being a ‘trigger’ for cardiovascular events (Sekine et al., 2010). Our study did not consider how interactions between individual differences in circadian phase and sleep restriction may affect vascular function. However, future work may heed associations between chronotype and diurnal variations in endothelial function (Facer‐Childs et al., 2019) to standardize the putative interactions between circadian phase and vascular function during sleep restriction. Last, future investigations should examine participants during both the morning and evening after sleep restriction, given previous reports that indicate diurnal differences in the circulatory milieu after chronic sleep restriction (McAlpine et al., 2022).

### 4.2 Conclusion

Acute PSR did not affect measures of arterial structure or function. We found no association between sleep duration and BA FMD, and this result persisted even after adjusting for arterial diameter and the shear rate dilatory stimulus. Cardiorespiratory fitness estimated by relative ${\dot{V}}_{O_{2}\mathrm{peak}}$ does not seem to play a role in the haemodynamic response to acute PSR in healthy young adults. Exploratory *ex vivo* analyses accorded with the data collected in vivo, and revealed no indication that intracellular processes involved in circadian rhythms or endothelial function were significantly affected during acute PSR. Our results add to a growing body of literature indicating that while the endothelium is vulnerable to prolonged instances of both partial (Shah et al. 2022) and total sleep restriction (Holmer et al., 2021), it is resilient after acute instances of partial sleep restriction.

## AUTHOR CONTRIBUTIONS

Joshua M. Cherubini, Jem L. Cheng, and Maureen J. MacDonald conceptualized and designed experiments; Joshua M. Cherubini, Jem L. Cheng, Calvin M. Armstrong, and Michael J. Kamal collected data and performed experiments; Joshua M. Cherubini analysed the data; Joshua M. Cherubini prepared an initial draft of the manuscript, which was edited by all co‐authors. Gianni Parise and Maureen J. MacDonald supervised, directed, and managed the study. All authors have read and approved the final version of this manuscript and agree to be accountable for all aspects of the work in ensuring that questions related to the accuracy or integrity of any part of the work are appropriately investigated and resolved. All persons designated as authors qualify for authorship, and all those who qualify for authorship are listed.

## CONFLICT OF INTEREST

No competing interests, financial or otherwise, are declared by the authors.

## Data Availability

The data and analyses to support these findings are publicly available in the Borealis data repository: https://doi.org/10.5683/SP3/9HFTTW

## References

[eph13585-bib-0001] Ai, S. , Zhang, J. , Zhao, G. , Wang, N. , Li, G. , So, H. C. , Liu, Y. , Chau, S. W. , Chen, J. , Tan, X. , & Jia, F. (2021). Causal associations of short and long sleep durations with 12 cardiovascular diseases: Linear and nonlinear Mendelian randomization analyses in UK Biobank. European Heart Journal, 42(34), 3349–3357. 33822910 10.1093/eurheartj/ehab170

[eph13585-bib-0002] Balsalobre, A. , Damiola, F. , & Schibler, U. (1998). A serum shock induces circadian gene expression in mammalian tissue culture cells. Cell, 93(6), 929–937. 9635423 10.1016/s0092-8674(00)81199-x

[eph13585-bib-0003] Brunt, V. E. , Weidenfeld‐Needham, K. M. , Comrada, L. N. , Francisco, M. A. , Eymann, T. M. , & Minson, C. T. (2019). Serum from young, sedentary adults who underwent passive heat therapy improves endothelial cell angiogenesis via improved nitric oxide bioavailability. Temperature, 6(2), 169–178. 10.1080/23328940.2019.1614851 PMC6601412 31286027

[eph13585-bib-0004] Caldwell, A. R. (2022). Exploring equivalence testing with the updated TOSTER R package. Psyarxiv. https://aaroncaldwell.us/TOSTERpkg

[eph13585-bib-0005] Camm, A. J. , Malik, M. , Bigger, J. T. , Breithardt, G. , Cerutti, S. , Cohen, R. J. , Coumel, P. , Fallen, E. L. , Kennedy, H. L. , Kleiger, R. E. , & Lombardi, F. (1996). Heart rate variability: Standards of measurement, physiological interpretation and clinical use. Task Force of the European Society of Cardiology and the North American Society of Pacing and Electrophysiology. Circulation, 93(5), 1043–1065. 8598068

[eph13585-bib-0006] Carney, C. E. , Buysse, D. J. , Ancoli‐Israel, S. , Edinger, J. D. , Krystal, A. D. , Lichstein, K. L. , & Morin, C. M. (2012). The consensus sleep diary: Standardizing prospective sleep self‐monitoring. Sleep, 35(2), 287–302. 22294820 10.5665/sleep.1642 PMC3250369

[eph13585-bib-0007] Cedernaes, J. , Osler, M. E. , Voisin, S. , Broman, J. E. , Vogel, H. , Dickson, S. L. , Zierath, J. R. , Schiöth, H. B. , & Benedict, C. (2015). Acute sleep loss induces tissue‐specific epigenetic and transcriptional alterations to circadian clock genes in men. The Journal of Clinical Endocrinology & Metabolism, 100(9), E1255–E1261. 26168277 10.1210/JC.2015-2284

[eph13585-bib-0008] Celermajer, D. S. , Sorensen, K. E. , Gooch, V. M. , Spiegelhalter, D. J. , Miller, O. I. , Sullivan, I. D. , Lloyd, J. K. , & Deanfield, J. (1992). Non‐invasive detection of endothelial dysfunction in children and adults at risk of atherosclerosis. The Lancet, 340(8828), 1111–1115. 10.1016/0140-6736(92)93147-f 1359209

[eph13585-bib-0009] Cherubini, J. M. , Cheng, J. L. , Williams, J. S. , & MacDonald, M. J. (2021). Sleep deprivation and endothelial function: Reconciling seminal evidence with recent perspectives. American Journal of Physiology‐Heart and Circulatory Physiology, 320(1), H29–H35. 33064569 10.1152/ajpheart.00607.2020

[eph13585-bib-0010] Cherubini, J. M. , & MacDonald, M. J. (2021). Statistical inferences using effect sizes in human endothelial function research. Artery Research, 27(4), 176–185. 34966462 10.1007/s44200-021-00006-6 PMC8654719

[eph13585-bib-0011] Covassin, N. , Bukartyk, J. , Singh, P. , Calvin, A. D. , St Louis, E. K. , & Somers, V. K. (2021). Effects of experimental sleep restriction on ambulatory and sleep blood pressure in healthy young adults: A randomized crossover study. Hypertension, 78(3), 859–870. 34247512 10.1161/HYPERTENSIONAHA.121.17622 PMC8363516

[eph13585-bib-0012] Craighead, D. H. , Heinbockel, T. C. , Freeberg, K. A. , Rossman, M. J. , Jackman, R. A. , Jankowski, L. R. , Hamilton, M. N. , Ziemba, B. P. , Reisz, J. A. , D'Alessandro, A. , & Brewster, L. M. (2021). Time‐efficient inspiratory muscle strength training lowers blood pressure and improves endothelial function, NO bioavailability, and oxidative stress in midlife/older adults with above‐normal blood pressure. Journal of the American Heart Association, 10(13), e020980. 34184544 10.1161/JAHA.121.020980 PMC8403283

[eph13585-bib-0013] Daghlas, I. , Dashti, H. S. , Lane, J. , Aragam, K. G. , Rutter, M. K. , Saxena, R. , & Vetter, C. (2019). Sleep duration and myocardial infarction. Journal of the American College of Cardiology, 74(10), 1304–1314. 31488267 10.1016/j.jacc.2019.07.022 PMC6785011

[eph13585-bib-0014] Dettoni, J. L. , Consolim‐Colombo, F. M. , Drager, L. F. , Rubira, M. C. , Cavasin de Souza, S. B. , Irigoyen, M. C. , Mostarda, C. , Borile, S. , Krieger, E. M. , Moreno, Jr. H. , & Lorenzi‐Filho, G. (2012). Cardiovascular effects of partial sleep deprivation in healthy volunteers. Journal of Applied Physiology, 113(2), 232–236. 22539169 10.1152/japplphysiol.01604.2011

[eph13585-bib-0015] Engert, L. C. , Mullington, J. M. , & Haack, M. (2023). Prolonged experimental sleep disturbance affects the inflammatory resolution pathways in healthy humans. Brain, Behavior, and Immunity, 113, 12–20. 37369338 10.1016/j.bbi.2023.06.018 PMC10528069

[eph13585-bib-0016] Facer‐Childs, E. R. , Pake, K. , Lucas, S. J. , & Balanos, G. M. (2019). Diurnal variations in vascular endothelial vasodilation are influenced by chronotype in healthy humans. Frontiers in Physiology, 10, 447329. 10.3389/fphys.2019.00901 PMC6635887 31354532

[eph13585-bib-0017] Full, K. M. , Huang, T. , Shah, N. A. , Allison, M. A. , Michos, E. D. , Duprez, D. A. , Redline, S. , & Lutsey, P. L. (2023). Sleep irregularity and subclinical markers of cardiovascular disease: The multi‐ethnic study of atherosclerosis. Journal of the American Heart Association, 12(4), e027361. 36789869 10.1161/JAHA.122.027361 PMC10111477

[eph13585-bib-0018] Furchgott, R. F. , & Zawadzki, J. V. (1980). The obligatory role of endothelial cells in the relaxation of arterial smooth muscle by acetylcholine. Nature, 288(5789), 373–376. 6253831 10.1038/288373a0

[eph13585-bib-0019] Han, M. , Qie, R. , Shi, X. , Yang, Y. , Lu, J. , Hu, F. , Zhang, M. , Zhang, Z. , Hu, D. , & Zhao, Y. (2022). Cardiorespiratory fitness and mortality from all causes, cardiovascular disease and cancer: Dose–response meta‐analysis of cohort studies. British Journal of Sports Medicine, 56(13), 733–739. 35022163 10.1136/bjsports-2021-104876

[eph13585-bib-0020] Herbert, S. P. , Odell, A. F. , Ponnambalam, S. , & Walker, J. H. (2009). Activation of cytosolic phospholipase A2‐α as a novel mechanism regulating endothelial cell cycle progression and angiogenesis. Journal of Biological Chemistry, 284(9), 5784–5796. 19119141 10.1074/jbc.M807282200 PMC2645829

[eph13585-bib-0021] Holmer, B. J. , Lapierre, S. S. , Jake‐Schoffman, D. E. , & Christou, D. D. (2021). Effects of sleep deprivation on endothelial function in adult humans: A systematic review. Geroscience, 43(1), 137–158. 33558966 10.1007/s11357-020-00312-y PMC8050211

[eph13585-bib-0022] Howley, E. T. , Bassett, D. R. , & Welch, H. G. (1995). Criteria for maximal oxygen uptake: Review and commentary. Medicine & Science in Sports & Exercise, 27(9), 1292–1301. 8531628

[eph13585-bib-0023] Ignarro, L. J. , Buga, G. M. , Wood, K. S. , Byrns, R. E. , & Chaudhuri, G. (1987). Endothelium‐derived relaxing factor produced and released from artery and vein is nitric oxide. Proceedings of the National Academy of Sciences, 84(24), 9265–9269. 10.1073/pnas.84.24.9265 PMC299734 2827174

[eph13585-bib-0024] Inaba, Y. , Chen, J. A. , & Bergmann, S. R. (2010). Prediction of future cardiovascular outcomes by flow‐mediated vasodilatation of brachial artery: A meta‐analysis. The International Journal of Cardiovascular Imaging, 26, 631–640. 20339920 10.1007/s10554-010-9616-1

[eph13585-bib-0025] Irwin, M. R. (2019). Sleep and inflammation: Partners in sickness and in health. Nature Reviews Immunology, 19(11), 702–715. 10.1038/s41577-019-0190-z 31289370

[eph13585-bib-0026] Irwin, M. R. , Olmstead, R. , & Carroll, J. E. (2016). Sleep disturbance, sleep duration, and inflammation: A systematic review and meta‐analysis of cohort studies and experimental sleep deprivation. Biological Psychiatry, 80(1), 40–52. 26140821 10.1016/j.biopsych.2015.05.014 PMC4666828

[eph13585-bib-0027] Irwin, M. R. , Wang, M. , Campomayor, C. O. , Collado‐Hidalgo, A. , & Cole, S. (2006). Sleep deprivation and activation of morning levels of cellular and genomic markers of inflammation. Archives of Internal Medicine, 166(16), 1756–1762. 16983055 10.1001/archinte.166.16.1756

[eph13585-bib-0028] Jiang, J. , Gan, Z. , Li, Y. , Zhao, W. , Li, H. , Zheng, J. P. , & Ke, Y. (2017). REM sleep deprivation induces endothelial dysfunction and hypertension in middle‐aged rats: Roles of the eNOS/NO/cGMP pathway and supplementation with L‐arginine. PLoS ONE, 12(8), e0182746. 28809932 10.1371/journal.pone.0182746 PMC5557538

[eph13585-bib-0029] Jones, H. , Green, D. J. , George, K. , & Atkinson, G. (2010). Intermittent exercise abolishes the diurnal variation in endothelial‐dependent flow‐mediated dilation in humans. American Journal of Physiology‐Regulatory, Integrative and Comparative Physiology, 298(2), R427–R432. 19923362 10.1152/ajpregu.00442.2009

[eph13585-bib-0030] Jones, H. , Lewis, N. C. , Thompson, A. , Marrin, K. , Green, D. J. , & Atkinson, G. (2012). Diurnal variation in vascular function: Role of sleep. Chronobiology International, 29(3), 271–277. 22390240 10.3109/07420528.2012.654554

[eph13585-bib-0031] Lakens, D. (2017). Equivalence tests: A practical primer for t tests, correlations, and meta‐analyses. Social Psychological and Personality Science, 8(4), 355–362. 28736600 10.1177/1948550617697177 PMC5502906

[eph13585-bib-0032] Lakens, D. , & Caldwell, A. R. (2021). Simulation‐based power analysis for factorial analysis of variance designs. Advances in Methods and Practices in Psychological Science, 4(1), 2515245920951503.

[eph13585-bib-0033] LaRocca, T. J. , Henson, G. D. , Thorburn, A. , Sindler, A. L. , Pierce, G. L. , & Seals, D. R. (2012). Translational evidence that impaired autophagy contributes to arterial ageing. The Journal of Physiology, 590(14), 3305–3316. 22570377 10.1113/jphysiol.2012.229690 PMC3459044

[eph13585-bib-0064] Livak, K. J. , & Schmittgen, T. D. (2001). Analysis of relative gene expression data using real‐time quantitative PCR and the 2− ΔΔCT method. Methods, 25(4), 402–408. 11846609 10.1006/meth.2001.1262

[eph13585-bib-0034] Lund, H. G. , Reider, B. D. , Whiting, A. B. , & Prichard, J. R. (2010). Sleep patterns and predictors of disturbed sleep in a large population of college students. Journal of Adolescent Health, 46(2), 124–132. 10.1016/j.jadohealth.2009.06.016 20113918

[eph13585-bib-0036] Mastronardi, C. A. , Yu, W. H. , & McCann, S. M. (2002). Resting and circadian release of nitric oxide is controlled by leptin in male rats. Proceedings of the National Academy of Sciences, 99(8), 5721–5726. 10.1073/pnas.082098499 PMC122838 11960027

[eph13585-bib-0037] Mastrullo, V. , van der Veen, D. R. , Gupta, P. , Matos, R. S. , Johnston, J. D. , McVey, J. H. , Madeddu, P. , Velliou, E. G. , & Campagnolo, P. (2022). Pericytes’ circadian clock affect endothelial cells’ synchronization and angiogenesis in a 3D tissue engineered scaffold. Frontiers in Pharmacology, 13, 785. 10.3389/fphar.2022.867070 PMC8977840 35387328

[eph13585-bib-0038] McAlpine, C. S. , Kiss, M. G. , Rattik, S. , He, S. , Vassalli, A. , Valet, C. , Anzai, A. , Chan, C. T. , Mindur, J. E. , Kahles, F. , & Poller, W. C. (2019). Sleep modulates haematopoiesis and protects against atherosclerosis. Nature, 566(7744), 383–387. 30760925 10.1038/s41586-019-0948-2 PMC6442744

[eph13585-bib-0039] McAlpine, C. S. , Kiss, M. G. , Zuraikat, F. M. , Cheek, D. , Schiroli, G. , Amatullah, H. , Huynh, P. , Bhatti, M. Z. , Wong, L. P. , Yates, A. G. , & Poller, W. C. (2022). Sleep exerts lasting effects on hematopoietic stem cell function and diversity. Journal of Experimental Medicine, 219(11), e20220081. 36129517 10.1084/jem.20220081 PMC9499822

[eph13585-bib-0040] Mirabella, G. , Fragola, M. , Giannini, G. , Modugno, N. , & Lakens, D. (2017). Inhibitory control is not lateralized in Parkinson's patients. Neuropsychologia, 102, 177–189. 28647437 10.1016/j.neuropsychologia.2017.06.025

[eph13585-bib-0065] Montoya, A. K. (2019). Moderation analysis in two‐instance repeated measures designs: Probing methods and multiple moderator models. Behavior Research Methods, 51, 61–82. 30306409 10.3758/s13428-018-1088-6 PMC6420436

[eph13585-bib-0041] Morishima, T. , Tsuchiya, Y. , Ueda, H. , Tsuji, K. , & Ochi, E. (2020). Sitting‐induced endothelial dysfunction is prevented in endurance‐trained individuals. Medicine & Science in Sports & Exercise, 52(8), 1770–1775. 32079922 10.1249/MSS.0000000000002302

[eph13585-bib-0042] Otto, M. E. , Svatikova, A. , Barretto, R. B. , Santos, S. , Hoffmann, M. , Khandheria, B. , & Somers, V. (2004). Early morning attenuation of endothelial function in healthy humans. Circulation, 109(21), 2507–2510. 15136499 10.1161/01.CIR.0000128207.26863.C4

[eph13585-bib-0063] Papadakis, Z. , Forsse, J. S. , & Peterson, M. N. (2020). Acute partial sleep deprivation and high‐intensity interval exercise effects on postprandial endothelial function. European Journal of Applied Physiology, 120, 2431–2444. 32803383 10.1007/s00421-020-04468-5

[eph13585-bib-0043] Parsons, R. , Parsons, R. , Garner, N. , Oster, H. , & Rawashdeh, O. (2020). CircaCompare: A method to estimate and statistically support differences in mesor, amplitude and phase, between circadian rhythms. Bioinformatics, 36(4), 1208–1212. 31588519 10.1093/bioinformatics/btz730

[eph13585-bib-0044] Pyke, K. E. , & Tschakovsky, M. E. (2007). Peak vs. total reactive hyperemia: Which determines the magnitude of flow‐mediated dilation? Journal of Applied Physiology, 102(4), 1510–1519. 17170205 10.1152/japplphysiol.01024.2006

[eph13585-bib-0045] Sauvet, F. , Arnal, P. J. , Tardo‐Dino, P. E. , Drogou, C. , Van Beers, P. , Bougard, C. , Rabat, A. , Dispersyn, G. , Malgoyre, A. , Leger, D. , & Gomez‐Merino, D. (2017). Protective effects of exercise training on endothelial dysfunction induced by total sleep deprivation in healthy subjects. International Journal of Cardiology, 232, 76–85. 28089456 10.1016/j.ijcard.2017.01.049

[eph13585-bib-0046] Sauvet, F. , Drogou, C. , Bougard, C. , Arnal, P. J. , Dispersyn, G. , Bourrilhon, C. , Rabat, A. , Van Beers, P. , Gomez‐Merino, D. , Faraut, B. , & Leger, D. (2015). Vascular response to 1 week of sleep restriction in healthy subjects. A metabolic response? International Journal of Cardiology, 190, 246–255. 25932797 10.1016/j.ijcard.2015.04.119

[eph13585-bib-0047] Sauvet, F. , Leftheriotis, G. , Gomez‐Merino, D. , Langrume, C. , Drogou, C. , Van Beers, P. , Bourrilhon, C. , Florence, G. , & Chennaoui, M. (2010). Effect of acute sleep deprivation on vascular function in healthy subjects. Journal of Applied Physiology, 108(1), 68–75. 19910332 10.1152/japplphysiol.00851.2009

[eph13585-bib-0048] Schlagintweit, J. , Laharnar, N. , Glos, M. , Zemann, M. , Demin, A. V. , Lederer, K. , Penzel, T. , & Fietze, I. (2023). Effects of sleep fragmentation and partial sleep restriction on heart rate variability during night. Scientific Reports, 13(1), 6202. 37069226 10.1038/s41598-023-33013-5 PMC10110519

[eph13585-bib-0049] Sekine, T. , Daimon, M. , Hasegawa, R. , Toyoda, T. , Kawata, T. , Funabashi, N. , & Komuro, I. (2010). The impact of sleep deprivation on the coronary circulation. International Journal of Cardiology, 144(2), 266–267. 19203808 10.1016/j.ijcard.2009.01.013

[eph13585-bib-0050] Shah, R. , Emin, M. , Wei, Y. , Sampogna, R. , & Jelic, S. (2018). Sleep restriction increases endothelial oxidative stress and inflammation in women. Circulation, 138, (Suppl_1), A10898.

[eph13585-bib-0051] Shah, R. , St‐Onge, M. P. , Emin, M. , Gao, S. , Sampogna, R. V. , Aggarwal, B. , Wei, Y. , & Jelic, S. (2022). Sleep deprivation impairs vascular function in healthy women: A clinical trial. Annals of the American Thoracic Society, 19(12), 2097–2100. 36112109 10.1513/AnnalsATS.202205-406RL PMC9743480

[eph13585-bib-0052] Shang, X. , Pati, P. , Anea, C. B. , Fulton, D. J. , & Rudic, R. D. (2016). Differential regulation of BMAL1, CLOCK, and endothelial signaling in the aortic arch and ligated common carotid artery. Journal of Vascular Research, 53(5‐6), 269–278. 27923220 10.1159/000452410 PMC5765856

[eph13585-bib-0053] Stockelman, K. A. , Bain, A. R. , Dow, C. A. , Diehl, K. J. , Greiner, J. J. , Stauffer, B. L. , & DeSouza, C. A. (2021). Regular aerobic exercise counteracts endothelial vasomotor dysfunction associated with insufficient sleep. American Journal of Physiology‐Heart and Circulatory Physiology, 320(3), H1080–H1088. 33416458 10.1152/ajpheart.00615.2020 PMC7988760

[eph13585-bib-0054] Takase, B. , Uehata, A. , Akima, T. , Nagai, T. , Nishioka, T. , Hamabe, A. , Satomura, K. , Ohsuzu, F. , & Kurita, A. (1998). Endothelium‐dependent flow‐mediated vasodilation in coronary and brachial arteries in suspected coronary artery disease. American Journal of Cardiology, 82(12), 1535–1539. 9874063 10.1016/s0002-9149(98)00702-4

[eph13585-bib-0055] Takeda, N. , Maemura, K. , Horie, S. , Oishi, K. , Imai, Y. , Harada, T. , Saito, T. , Shiga, T. , Amiya, E. , Manabe, I. , & Ishida, N. (2007). Thrombomodulin is a clock‐controlled gene in vascular endothelial cells. Journal of Biological Chemistry, 282(45), 32561–32567. 17848551 10.1074/jbc.M705692200

[eph13585-bib-0056] Thijssen, D. H. , Bruno, R. M. , van Mil, A. C. , Holder, S. M. , Faita, F. , Greyling, A. , Zock, P. L. , Taddei, S. , Deanfield, J. E. , Luscher, T. , & Green, D. J. (2019). Expert consensus and evidence‐based recommendations for the assessment of flow‐mediated dilation in humans. European Heart Journal, 40(30), 2534–2547. 31211361 10.1093/eurheartj/ehz350

[eph13585-bib-0057] Thosar, S. S. , Berman, A. M. , Herzig, M. X. , McHill, A. W. , Bowles, N. P. , Swanson, C. M. , Clemons, N. A. , Butler, M. P. , Clemons, A. A. , Emens, J. S. , & Shea, S. A. (2019). Circadian rhythm of vascular function in midlife adults. Arteriosclerosis, Thrombosis, and Vascular Biology, 39(6), 1203–1211. 31070470 10.1161/ATVBAHA.119.312682 PMC6531330

[eph13585-bib-0058] Van Bortel, L. M. , Laurent, S. , Boutouyrie, P. , Chowienczyk, P. , Cruickshank, J. K. , de Backer, T. , Filipovsky, J. , Huybrechts, S. , Mattace‐Raso, F. U. , Protogerou, A. D. , & Schillaci, G. (2012). Expert consensus document on the measurement of aortic stiffness in daily practice using carotid‐femoral pulse wave velocity. Journal of Hypertension, 30(3), 445–448. 22278144 10.1097/HJH.0b013e32834fa8b0

[eph13585-bib-0059] Yang, H. , Baltzis, D. , Bhatt, V. , Haack, M. , Meier‐Ewert, H. K. , Gautam, S. , Veves, A. , & Mullington, J. M. (2021). Macro‐and microvascular reactivity during repetitive exposure to shortened sleep: Sex differences. Sleep, 44(5), zsaa257. 33249482 10.1093/sleep/zsaa257 PMC8120341

[eph13585-bib-0060] Yang, H. , Durocher, J. J. , Larson, R. A. , DellaValla, J. P. , & Carter, J. R. (2012). Total sleep deprivation alters cardiovascular reactivity to acute stressors in humans. Journal of Applied Physiology, 113(6), 903–908. 22815387 10.1152/japplphysiol.00561.2012 PMC3472479

